# Increased Vertical Impact Forces and Altered Running Mechanics with Softer Midsole Shoes

**DOI:** 10.1371/journal.pone.0125196

**Published:** 2015-04-21

**Authors:** Jennifer Baltich, Christian Maurer, Benno M. Nigg

**Affiliations:** Human Performance Laboratory (HPL), Faculty of Kinesiology, University of Calgary, Calgary, Alberta, Canada; University of Zaragoza, SPAIN

## Abstract

To date it has been thought that shoe midsole hardness does not affect vertical impact peak forces during running. This conclusion is based partially on results from experimental data using homogeneous samples of participants that found no difference in vertical impact peaks when running in shoes with different midsole properties. However, it is currently unknown how apparent joint stiffness is affected by shoe midsole hardness. An increase in apparent joint stiffness could result in a harder landing, which should result in increased vertical impact peaks during running. The purpose of this study was to quantify the effect of shoe midsole hardness on apparent ankle and knee joint stiffness and the associated vertical ground reaction force for age and sex subgroups during heel-toe running. 93 runners (male and female) aged 16-75 years ran at 3.33 ± 0.15 m/s on a 30 m-long runway with soft, medium and hard midsole shoes. The vertical impact peak increased as the shoe midsole hardness decreased (mean(SE); soft: 1.70BW(0.03), medium: 1.64BW(0.03), hard: 1.54BW(0.03)). Similar results were found for the apparent ankle joint stiffness where apparent stiffness increased as the shoe midsole hardness decreased (soft: 2.08BWm/º x 100 (0.05), medium: 1.92 BWm/º x 100 (0.05), hard: 1.85 BWm/º x 100 (0.05)). Apparent knee joint stiffness increased for soft (1.06BWm/º x 100 (0.04)) midsole compared to the medium (0.95BWm/º x 100 (0.04)) and hard (0.96BWm/º x 100 (0.04)) midsoles for female participants. The results from this study confirm that shoe midsole hardness can have an effect on vertical impact force peaks and that this may be connected to the hardness of the landing. The results from this study may provide useful information regarding the development of cushioning guidelines for running shoes.

## Introduction

Impact forces during heel-toe running have been discussed in the scientific literature for many years. Some have argued that increased impact forces are associated with the development of specific running injuries [1–4]. As a result, the development of shoe cushioning guidelines evolved as repetitive loading became a concern for injury risk during running. Several strategies have been proposed to reduce impact loading. One of the most popular approaches was to change the hardness of the shoe midsole [5–8]. However, this strategy was associated with the surprising result that in many studies, shoe midsole hardness had little to no effect on impact force peaks during landing [5–7]. This result is surprising because it was assumed that one could easily reduce the impact force peaks with soft shoe midsoles. However, the results of many initial studies have shown that this is not the case [5–7]. It may be that the results have been influenced by typically homogeneous test subject groups, which were mostly young sporty male university students. For this well-trained population, shoe midsole hardness may not be enough to influence running style. It may be that the result would be different for less trained or for elderly subjects [5–7]. However, the influence of shoe midsole hardness on impact loading during over ground running across different age and sex subgroups has yet to be examined.

Another possible reason for the lack of change in impact forces found with midsole hardness may be that subjects change something else during the initial ground contact in a softer shoe. There are different candidates for this change. One is to change the landing velocity when changing the hardness of the midsole. Another is to change the stride length [9–11]. However, it has been shown that there is no support for these two possible interpretations [12,13]. Another possibility is that subjects adjust their landing mechanics when running in different midsole shoes, changing their effective mass during landing by making their ankle and/or knee joint more stiff when the shoe sole becomes softer [14–16]. A stiffer limb would result in a “harder” landing (higher impact forces), counteracting any influence of the shoe cushioning on impact absorption. This has been supported by previous studies that have demonstrated increased leg or joint stiffness when running or hopping on a softer surface or a shoe with a thicker midsole [13, 16–19]. One method to quantify the hardness of a landing is to determine the relationship between the landing kinematics and kinetics, or the apparent stiffness of the joint [15, 20, 21]. This will provide an indication of how much joint displacement occurs for a given external force (moment) at the joint. The influence of shoe midsole cushioning on landing mechanics and the hardness of the landing may differ across age and gender subgroups. Landing mechanics can be influenced by a variety of factors, including lower extremity kinematics, the active forces of the muscles and the external forces applied to the body. Previous studies have demonstrated that age, sex, and footwear influence these factors [22–25]. Therefore, it may be speculated that the influence of shoe midsole hardness on landing mechanics may change across gender and sex subgroups.

While landing kinematics and kinetics have been investigated independently when running in shoes with different midsole properties, the relationship between the two has yet to be quantified. Understanding how shoe midsole hardness influences ankle and knee joint apparent stiffness and the resulting vertical impact peaks during heel-toe running and whether or not this influence differs across age and sex subgroups will provide useful information for the understanding of the landing during heel-toe running. This understanding would provide a small but significant addition to the understanding of the control strategies of the human system. The human system adapts as the boundary condition (e.g. footwear) changes. Investigating the different adaptation processes across a wide age range could shed some light onto the control strategies of the human system. Understanding these landing mechanisms due to shoe properties may allow for the construction of footwear that ultimately optimizes the running pattern or reduces the incidence of injuries.

Therefore, the purpose of this study was to characterize the effect of shoe midsole hardness on the apparent joint stiffness at the ankle and the knee and the resulting vertical ground reaction force during heel-toe running for different age and sex subgroups.

Based on previous results for differences in kinematics and muscular strength across the tested groups [22–25], it was hypothesized that:

(H1) Apparent ankle joint stiffness increases as shoe midsole hardness decreases for all age and sex groups.
(H2) Apparent knee joint stiffness will not be different between the shoe conditions.
(H3) Vertical impact force peaks will not be different between shoe conditions.

## Methods

### Subjects

Ninety-three recreational runners (47 male, 46 female) who ran at least 30 minutes per week participated in this study (Table 1). All participants provided written informed consent in accordance with the University of Calgary’s policy on research using human subjects and approval for this research project was obtained from the University of Calgary’s Conjoint Health Research Ethics Board. Written informed consent was provided by the legal guardian of the minors that participated in this study. All subjects were free from injury or pain at the time of testing. Four age groups ranging across the lifespan development stages were defined as follows: Group 1 (G1, adolescence) 16–20 years; Group 2 (G2, early adulthood) 21–35 years; Group 3 (G3, middle age) 36–60 years; and Group 4 (G4, older age) 61–75 years.

**Table 1:** Subject characteristics.

			Age		Height		Mass	
	Age Group	Number of Subjects	Average	SEM	Average	SEM	Average	SEM
Male	16–20	13	17.9	0.5	178.0	1.6	69.6	2.5
	21–35	13	25.5	1.1	179.7	2.0	74.0	2.0
	36–60	11	48.5	1.8	175.3	1.3	77.5	2.0
	61–75	10	66.9	1.5	174.5	1.8	74.6	3.0
Female	16–20	12	18.1	0.4	162.9	2.0	55.4	1.7
	21–35	12	26.2	0.9	166.8	2.4	62.5	2.1
	36–60	11	49.6	1.5	165.2	1.4	64.5	2.6
	61–75	11	65.5	1.5	162.0	1.6	55.6	1.5

Subject characteristics for each sex and age subgroup used in this study.

### Experimental setup

**Fig 1:**
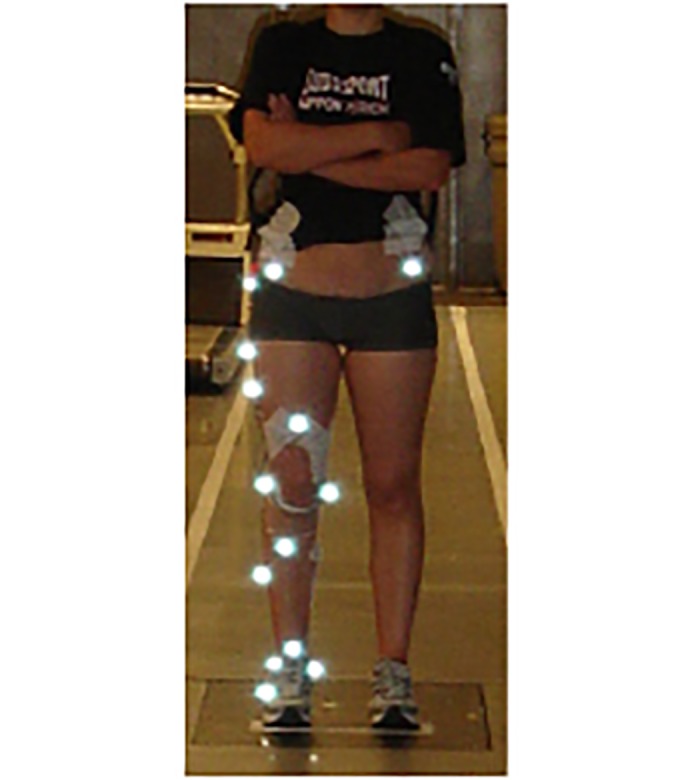
Marker Set Up. Frontal view of retro-reflective marker placement on the shoe, shank, thigh and pelvis.

Three different shoe conditions provided by Decathlon (now Oxylane Group, France) that differed only in their midsole hardness were investigated: Asker C-40 (Soft), Asker C-52 (Medium) and Asker C-65 (Hard). The shoes were identical with respect to all other footwear properties besides the midsole hardness. Kinematic data were collected using 12 retro-reflective markers mounted on the pelvis and right lower extremity (Fig 1) to measure three-dimensional movements of each segment using an eight-camera, 240 Hz motion capture system (Motion Analysis, CA). Kinetic data was collected simultaneously using a Kistler force plate at a sampling frequency of 2400 Hz (Kistler Instruments AG, Winterthur, Switzerland) embedded within the laboratory floor. A static trial was taken with markers placed over the right greater trochanter, medial and lateral knee joint axis, and medial and lateral malleoli in order to define the joint centers. Position data was collected for a static neutral trial for each of the shoe-subject conditions in order to define the segment coordinate system. Joint center markers were removed for the running trials.

Subjects performed five successful heel-toe running trials (3.33±0.15 m/s) for each of the three different shoe conditions on a 30 m running lane in the lab. The order in which the shoes were tested was randomly selected for each subject. Subjects were allotted at least three practice trials for familiarization prior to data collection for each of the shoe conditions.

### Data analysis

Markers were identified and their three-dimensional coordinates were tracked using EVaRT Real Time software (Version 5.0.4, Motion Analysis, CA, USA). Data were filtered using a low pass fourth order Butterworth filter with a cutoff frequency of 12 Hz for the kinematic data and 100 Hz for the kinetic data. Joint angular displacements and resultant moments were calculated using the kinematic and ground reaction force data (Kintrak, HPL, University of Calgary). Specifically, ankle dorsiflexion/plantar flexion and knee flexion/extension angular displacement and resultant moments were calculated. All variables were clipped to the stance phase of the step with the right foot on the force plate with heel contact and toe-off determined using a 15 N threshold in the vertical ground reaction force.

**Fig 2:**
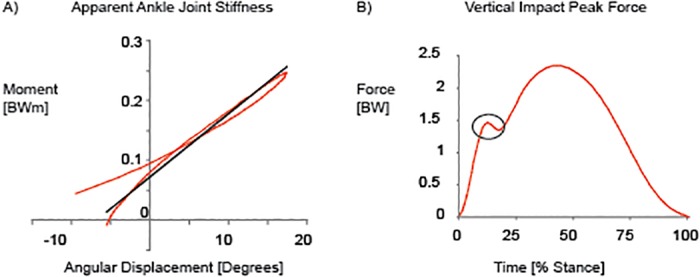
Methods. A) Illustration of apparent ankle joint stiffness (stiffness = slope), and B) vertical force during running highlighting the impact peak during the first part of stance.

The average apparent joint stiffness in the sagittal plane during the loading phase of stance for the ankle and knee joint were defined as the ratio of the change in joint moment (ΔM) to the joint angular displacement (Δθ) [18]. For the ankle, this corresponded to the change in ankle dorsiflexion/plantar flexion moment to the change in dorsiflexion/plantar flexion angular displacement. For the knee joint, this corresponded to the change in knee flexion/extension moment to the change in flexion/extension angular displacement. A least squares linear regression equation was used for the resultant joint-moment versus joint-angle curves for the loading portion of the stance phase (10%-50% of stance) and the slope of this line was identified as the apparent joint stiffness [20] (Fig 2A). The vertical impact force peak was calculated as the local maximum of the vertical ground reaction force during the first 50 ms following heel strike [26] (Fig 2B). A footwear condition was not included if an impact peak was not present in at least three of the five running trials.

A mixed model was used to perform a repeated measures ANCOVA (IBM SPSS Statistics 20.0, IL, USA) using a within subject factor of footwear (3 levels: soft, medium, and hard) and between subjects factors of age (4 levels: 16–20 years (n = 25), 21–35 years (n = 25), 36–60 years (n = 22), 61–75 years (n = 21)) and sex (2 levels: male (n = 47), female (n = 46)) for each of the three variables measured in this study in order to determine main effects and interaction effects. Height and weight were included as covariates. A pairwise comparison with a Bonferroni correction was then used if any significant main effects were found for footwear, age, or sex. All reported p-values are Bonferroni-corrected p-values and, thus, statistical significant was set at the α = 0.05 level.

## Results

**Fig 3:**
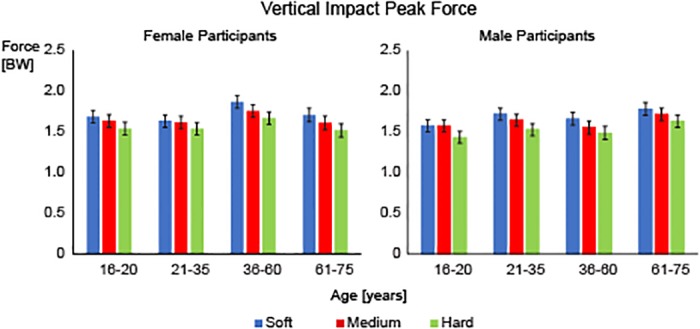
Vertical Impact Peak. Average vertical impact peak force (mean ± SEM) for the soft (blue), medium (red) and hard (green) midsole shoes for the female participants (left) and male participants (right). Significant differences were found between soft, medium and hard midsole shoes. Covariates were evaluated at the following values: weight = 66.7kg, Height = 170.9cm.

Descriptive statistics for the variables tested in this study can be seen in Table 2. There was a significant main effect for footwear for the vertical impact peak (F = 54.877, p<0.001). Post-hoc comparisons revealed that all footwear conditions were significantly different from each other (all p<0.001). The magnitude of the vertical impact peak increased as the shoe midsole hardness decreased with the soft midsole shoe having the largest vertical impact peak (mean (SE): 1.70BW (0.03)) followed by the medium midsole shoe (mean (SE): 1.64BW (0.03)) and finally the hard midsole shoe (mean (SE): 1.54BW (0.03)) (Fig 3).

**Table 2:** Statistics. Descriptive statistics (mean and standard deviation (SE)) for the variables tested.

Outcome Measure	Shoe Condition	Mean (SE)	Shoe Effect (F,[p])	Sex Effect (F,[p])	Age Effect (F,[p])	Weight Effect (F,[p])	Height Effect (F,[p])	Shoe Sex Interaction (F,[p])	Shoe Age Interaction {F,[p])	Sex Age Interaction (F,[p])	Shoe Sex Age Interaction (F,[p])
Apparent Ankle Joint Stiffness (BWm/◦ x 100)	Soft	2.08 (0.05)	55.409 [p<0.001]	0.174 [0.678]	0.545 [0.653]	0.957 [0.331]	4.067 [0.047]	2.155 [0.119]	1.060 [0.389]	1.454 [0.233]	1.142 [0.340]
	Medium	1.92 (0.05)									
	Hard	1.85 (0.05)									
Apparent Knee Joint Stiffness (BWm/◦ x 100)	Soft	0.96 (0.02)	26.254 [p<0.001]	6.260 [0.014]	1.574 [0.202]	0.891 [0.348]	13.593 [p<0.001]	6.336 [0.002]	1.138 [0.342]	0.970 [0.411]	0.338 [0.916]
	Medium	0.89 (0.02)									
	Hard	0.91 (0.02)									
Vertical Impact Peak(BW)	Soft	1.70 (0.03)	54.877 [p<0.001]	0.329 [0.568]	0.690 [0.561]	0.215 [0.644]	1.656 [0.202]	0.103 [0.903]	0.743 [0.616]	1.867 [0.142]	0.613 [0.720]
	Medium	1.64 (0.03)									
	Hard	1.54 (0.03)									

**Fig 4:**
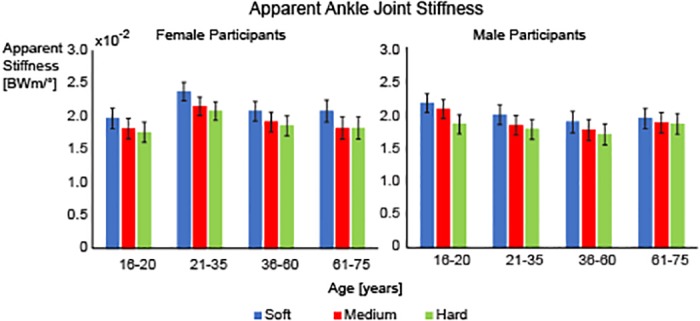
Apparent Ankle Stiffness. Average apparent ankle joint stiffness (mean ± SEM) for the soft (blue), medium (red) and hard (green) midsole shoes for the female participants (left) and male participants (right). Significant differences were found between soft, medium and hard midsole shoes. Covariates were evaluated at the following values: weight = 66.7kg, Height = 170.9cm.

There was a significant main effect for footwear for the apparent ankle joint stiffness (F = 55.409, p<0.001). Post-hoc comparisons revealed that all footwear conditions were significantly different from each other (all p≤0.001). Apparent ankle joint stiffness increased as shoe midsole hardness decreased. The average apparent stiffness during the loading phase of stance was highest for the soft midsole shoe (mean (SE): 2.08BWm/degrees x 100 (0.05)) followed by the medium midsole shoe (mean (SE): 1.92BWm/degrees x 100 (0.05)) and finally the hard midsole shoe (mean (SE): 1.85BWm/degrees x 100 (0.05)) (p<0.001) (Fig 4). There were no significant main effects for age or sex of the runner.

**Fig 5:**
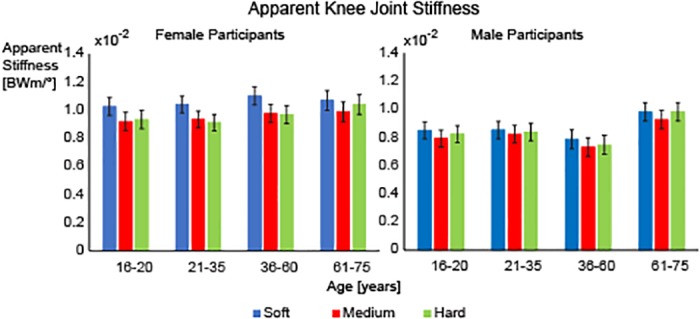
Apparent Knee Stiffness. Average apparent knee joint stiffness (mean ± SEM) for the soft (blue), medium (red) and hard (green) midsole shoes for the female participants (left) and male participants (right). Significant differences were found between the soft midsole and the medium and hard midsoles for the female participants and between the soft midsole and the medium midsole for the male participants. Covariates were evaluated at the following values: weight = 66.7kg, Height = 170.9cm.

Apparent knee joint stiffness showed a sex-dependent effect due to the shoe condition. There was a significant shoe sex interaction (F = 6.336, p = 0.002). For female subjects, the apparent knee joint stiffness increased in the soft midsole shoe (mean (SE): 1.06BWm/degrees x 100 (0.04)) compared to the medium midsole (mean (SE): 0.95BWm/degrees x 100 (0.04)) and the hard midsole shoe (mean (SE): 0.96BWm/degrees x 100 (0.04)) (both p<0.001). For male participants, there was a significant difference between the soft midsole shoe (mean (SE): 0.87BWm/degrees x 100 (0.04)) and the medium midsole shoe (mean (SE): 0.82BWm/degrees x 100 (0.04)) (p = 0.006) but there were no differences with respect to the hard midsole shoe (mean (SE): 0.85BWm/degrees x 100 (0.04)) (Fig 5).

## Discussion

The current study has two major results that are of specific interest with respect to impact forces. First, the study shows an effect of midsole hardness on vertical impact force peaks. Second, the study shows a potential connection between midsole hardness and apparent ankle/knee joint stiffness.

The influence of shoe midsole hardness on vertical impact peaks during running has been a question of high interest in running research [5–8]. Most studies report that there is no correlation between midsole hardness and vertical impact force peaks [6,7]. There is one study that indicates that the vertical impact force peaks increase for very soft and very hard midsole hardness [8]. However, these specific results have not been discussed in the literature. The results from the current study indicate that wearing soft midsole shoes can result in increased vertical impact force peaks. There are different possible interpretations of this result. First, it may be that the higher vertical impact force peaks are a result of “bottoming out” of the midsole. Second, it may be that the higher impact force peaks are a result of the increased apparent stiffness at the ankle and knee joints. Individuals in the present study adjusted their apparent joint stiffness when running in shoes with different midsole properties. Specifically, the majority of the tested individuals increased the apparent stiffness in their joints when running in softer shoes. The resulting increase in the vertical impact force peaks during landing could imply an increase of the loading on the tissues when running in softer midsole shoes.

Apparent joint stiffness was sensitive to the shoe midsole hardness, specifically at the ankle joint. The apparent ankle joint stiffness increased as the shoe midsole hardness decreased (Fig 4) and the effects were systematic across sex and age groups. Similar findings have been found when comparing barefoot and shod running where ankle stiffness was higher in the softer cushioned footwear condition compared to barefoot running [19]. Overall leg stiffness has also been shown to increase during softer cushioned footwear conditions compared to barefoot running [16]. It has been speculated previously that the apparent stiffness of the lower extremity increases as the demand of the activity increases, as seen through increased apparent joint stiffness with increasing running velocity [20]. Thus, one could speculate that greater apparent joint stiffness may be required in order to control the joint movements in a softer midsole. Increased apparent joint stiffness could increase the decelerated mass at impact, which may explain the increase of the vertical impact peak for the soft midsole shoe condition.

The influence of shoe midsole hardness on apparent knee joint stiffness depended on the sex of the runner. Specifically, apparent knee joint stiffness was increased in the soft midsole condition compared to the medium and the hard midsole condition for the female participants. For male participants, there was a significant difference between the soft and the medium midsole shoe but not with the hard midsole shoe. For the soft and medium midsole shoes, the female participants also had more apparent knee joint stiffness than the male participants. For the hard midsole shoe, there was no difference between the males and females with respect to apparent knee joint stiffness. Thus, female participants appear to be more sensitive at the knee joint to the midsole hardness conditions used in this study. This may be related to other biomechanical and neuromuscular differences that have been found between males and females including muscle strength, proprioception, and joint laxity [27–30]. Females have been shown to have reduced joint position sense, lower hamstring to quad strength ratios, increased muscular co-contraction prior to landing and increased joint laxity [27–30]. It has been speculated that the altered muscle activity and increased co-contraction prior to landing in females may be compensatory mechanisms to improve joint stability due to their proprioceptive deficits and increased joint laxity [31–33]. It may be that the softer midsole shoe conditions used in this study were in a range that also required greater apparent knee joint stiffness as a mechanism for the female participants to increase joint stability and control the movements at the joint.

The results from the apparent ankle and knee joint stiffness also indicate that the ankle joint is more sensitive than the knee joint to changes in shoe midsole properties. Similar results have been found previously when examining the influence of shoe midsole hardness on lower extremity kinematics and kinetics. For example, Hardin and colleagues examined kinematic adaptations at the ankle, knee, and hip joint due to shoe midsole hardness and found that footwear only influenced movements at the ankle joint [13]. Similar results were also found when using a vector-based approach [34]. Von Tscharner and colleagues used an iterative support vector machine in order to identify kinematic differences due to footwear with different midsole properties. They found that differences due to footwear were located at the ankle joint and that there were no differences at the knee or hip joint due to footwear with different midsole properties. These kinematic results support the current findings that footwear appears to influence the distal ankle joint more strongly than the proximal knee joint.

Certain limitations exist for this study. The models used for apparent joint stiffness are rather simplistic and provide only an approximation of stiffness. Apparent joint stiffness has been termed as “quasi-stiffness” as it is not a true mechanical representation of stiffness [35]. However, this measures provides an indication of how much joint displacement occurs for a given external force (moment) at the joint, which in turn provides information about the hardness of the landing during over ground running. In addition, while the overall sample for shoe effects was large, the individual age and subgroup samples were still relatively small.

### Concluding remarks

Shoe midsole properties used in this study had a significant influence on the local measurement of apparent joint stiffness. Shoe midsole hardness affected the distal ankle joint more than the proximal knee joint, where differences depended on sex of the runner. The increase in apparent joint stiffness may have resulted in an increase in the decelerated mass at landing, effectively increasing the impact peak magnitude in the softer midsole conditions. This study provides experimental evidence that shoe midsole hardness can in fact affect vertical force impact peaks during running. Even more importantly, the results from this study showed that softer midsole shoes can actually increase external vertical force impact peaks. This contradicts the popular belief that softer midsole shoes should reduce impact peaks during running. The results from this study may provide useful information regarding the development of cushioning guidelines for running shoes. The shoe midsole properties used in this study encompass the midsole stiffness properties of shoes currently on the market. However, in order to develop more specific guidelines for midsole cushioning, a larger study would need to be conducted in which a variety of midsole stiffness levels are tested in order to determine if there is an ideal level of shoe midsole hardness.

The results from this study may also have important implications for the loads imposed on certain tissues of the leg and foot while running in different shoe midsoles. The studies concentrating on the effects of impact forces on injuries can be grouped into studies that claim that impact forces are associated with the development of specific running injuries [1–4], studies that claim that there is no connection between impact forces and injury development [36, 37] and into a few studies that claim that subjects with high impact forces or loading rates are less likely to be injured in heel-toe running [38–40]. All of these impact-related injury studies have the shortcoming that they typically use small sample sizes of subjects and, therefore, that the results are not conclusive. Future studies would need to investigate whether the changes in local measures of apparent joint stiffness due to shoe midsole hardness are related to either changes in performance or changes in the risk of injuries. This would allow for shoe manufacturers to develop cushioning guidelines based on injury prevention requirements.

## Supporting Information

S1 File Supplementary Data. Mean vertical impact peak, apparent ankle stiffness, and apparent knee stiffness for the three midsole shoes (soft, medium, hard) for each of the 93 participants in this study as well as the participant age, height, and weight.
(XLSX)
